# The role of intervention timing and treatment modality in visual recovery following pituitary apoplexy: a systematic review and meta-analysis

**DOI:** 10.1007/s11060-024-04717-z

**Published:** 2024-11-06

**Authors:** Nolan J. Brown, Saarang Patel, Julian Gendreau, Mickey E. Abraham

**Affiliations:** 1 Department of Neurological Surgery, University of California-Irvine, 101 The City Dr S, Orange, CA 92868 USA; 2 Johns Hopkins Whiting School of Engineering, Baltimore, MD USA; 3 Department of Neurological Surgery, University of California, San Diego, CA USA

**Keywords:** Neurosurgery, Skull base, Pituitary apoplexy, Visual deficits, Hemorrhage, Infarct, Pituitary adenoma, Sellar mass

## Abstract

**Introduction:**

Pituitary apoplexy has historically been considered an emergent condition that necessitates surgical intervention when there is acute symptomatic onset. This potentially serious condition often occurs in the setting of an underlying adenoma, cystic lesion, or other sellar mass. When these mass lesions hemorrhage within the confined space of the sella turcica, the pituitary gland is subjected to hemorrhagic ischemia. Furthermore, critical neurovasculature in close proximity to the sella can sustain collateral damage. In the present study, we investigate whether early versus delayed surgical intervention (in terms of three timelines: before versus after 48 h, 72 h, and 7 days, respectively) results in differences in visual outcomes for patients experiencing pituitary apoplexy with acute onset neurological and/or neuro-opthalmic symptoms. Furthermore, we compare the efficacy of surgical decompression versus expectant management of this condition.

**Methods:**

Accordingly, we queried the PubMed, Scopus, and Embase databases in adherence to PRISMA guidelines. Quantitative meta-analysis was performed according to the Mantel–Haenszel method and forest plots were generated using Review Manager v5.4.* P*-values < 0.05 were defined as the threshold for statistical significance.

**Results:**

Twenty-nine studies remained eligible for review following initial search and screen, including 16 studies describing the role of intervention timing and 15 studies comparing intervention modality. Most patients presented with a visual deficit, and all patients underwent surgery – most commonly via the endoscopic endonasal (EEA) approach. Two hundred and twenty patients were included in the sub-analysis for the 7-day cutoff point. Furthermore, 81 patients underwent surgical decompression of the sella prior to 48 h, and 32 patients underwent surgical decompression between 48–72 h following presentation. Almost all patients exhibited improved vision post-decompression, including 19/19 patients (100%) in the post-72-h cohort. On meta-analysis using the Mantel–Haenszel method, there was a significant difference in vision outcomes in favor of patients who underwent surgical decompression before 7 days as compared to after seven days (OR 5.88, 95% CI [1.77, 19.60], I^2^ = 0%, *p* < 0.01). In a separate sub-analysis, there was a total of 288 patients across 15 studies comparing surgical versus conservative management of pituitary apoplexy. These management options proved equivocal on meta-analysis (*p* > 0.05).

**Conclusion:**

In the present study, timing of surgical intervention for pituitary apoplexy was predictive of visual function recovery only at the 7-day timepoint, as has been reported by previous studies. Ultimately, this suggests that pituitary apoplexy involving severe visual deficits or altered mental status is best addressed within the first seven days post-presentation, and that both surgery and conservative management can offer similar outcomes. When apoplexy is suspected, IV corticosteroids should be administered independent of acuity or severity to prevent secondary adrenal crisis. Subsequently, for patients presenting without severe visual or other neurological deficits, expectant management is recommended. Management should be patient-specific and dependent upon the severity of symptoms present at onset.

## Introduction

Pituitary apoplexy (PA) is a serious condition resulting from hemorrhage or infarction of a mass lesion within the pituitary gland [1]. In select cases, apoplexy leads to infarction and merits urgent medical resuscitation (e.g., IV corticosteroid replacement therapy) followed by surgical decompression [1]. Signs of meningismus or altered mental status may conflate varying causes of a vague clinical picture and potentially delay diagnosis [2]. The etiology of PA involves sudden expansion of a hemorrhagic mass within the sella turcica, with the mass typically being a non-functioning pituitary macroadenoma, but sometimes involving a normal pituitary gland or other mass lesion [3]. In patients with adenomatous lesions, the incidence of apoplexy is approximately 13% [3, 4].

With respect to evaluation, CT or MRI will reveal a hemorrhagic mass within the sella turcica or parasellar region that frequently distorts the anterior wall of the 3rd ventricle [2]. MRI is useful for detecting fresh bleeding, and DWI can show increased signal intensity representative of ischemic tissue – including the smallest of infarcts – within minutes of the apoplectic event (by comparison, T1 and T2 sequences may not demonstrate infarct until > 6 h post-apoplexy). Furthermore, CTA can also be useful for surgical planning to aid in delineation of surrounding vascular structures during surgical planning for macroadenomas. It is important to note that cerebral angiography has also been described as a precipitant of PA, and is not standard of care for diagnosis. Once a diagnosis of PA is confirmed, first line treatment for acutely symptomatic patients consists of rapid administration of IV corticosteroids and surgical decompression. Furthermore, steroids should be administered independent of the level of acuity or severity for any patient exhibiting neurologic deficits in whom apoplexy is suspected.

As an alternative to medical resuscitation + surgery, some have reported Level III evidence suggesting that medical management alone is suitable for all severities of PA and that most patients with PA do not require emergent surgical intervention [4, 5]. Regardless, a majority of studies suggest that surgical decompression will eventually be needed for patients with severe neuro-opthalmic deficits [6–8]. Surgical decompression is normally performed through the transsphenoidal EEA approach, which enables facile resection of the hemorrhagic tumor or cyst [2]. Interestingly, optimal timing of surgical intervention for pituitary apoplexy remains an unsettled matter of debate [9]. In most cases, surgery within seven days of onset is recommended, yet others recommend operation within the first 48–72 h. Further investigation of optimal timing therefore seems warranted. To this end, we performed a systematic review of the literature to shed light on optimal timing of surgical intervention for pituitary apoplexy [5, 6]. Additionally, we compared visual outcomes by assessing functional recovery by type of intervention (surgical versus conservative).

## Methods

### Search strategy

A comprehensive search of the PubMed, Scopus, and Embase databases was performed on August 16th, 2023 in accordance with PRISMA guidelines to identify 1) all primary literature (including prospective trials, retrospective cohort studies, and case series) assessing 2) timing of surgical intervention for PA or 3) comparing surgical versus conservative treatment modalities. Each database was queried using the following Boolean search term: (pituitary apoplexy) AND (surgery OR intervention OR vision).

### Selection criteria

Studies were selected for inclusion if they: 1) presented data regarding timing of surgical intervention for PA and the role timing and intervention modality may have played in outcomes of treatment, including 2) rates of resolution of neuro-ophthalmologic symptoms and postoperative visual function recovery rates. Furthermore, studies were included if they 3) presented primary data in the form of prospective or retrospective cohort studies or multi-institutional experiences with pituitary apoplexy. Studies were excluded if they did not present primary data or were not available in full text in English or a suitable translation (Tables 1 and 2).

**Table 1:** Demographic Characteristics of Included Studies, Time of Intervention

Study	Institution, Country	Sample Size (%, female)	Mean Age (range) of Cohort	Patients Presenting with Visual Deficit (n, %)	Intervention Time Point	Visual Recovery (n, %) at early and late intervention time point
[10]	Mayo Clinic, USA	37 (32%)	56.6 (20–83)	n/a	7 days	10/10 (100%) 11/12 (92%)
Randeva et al., 2001	Radcliffe Infirmary, UK	35(40%)	49.8(30–74)	25 (71%)	7 days	10/10 (100%) 10/15 (77%)
Agarwal et al., 2005	All India Institute of Medical Sciences, India	8(13%)	43 (25–60)	8(100%)	7 days	2/3(67%) 2/5(40%)
Imboden et al., 2005	University Hospital, Lausanne, Switzerland	7(0%)	48(29–66)	7(100%)	7 days	5/5(100%) 2/2(100%)
[11]	Newcastle University Teaching Hospitals, UK	27 (30%)	50.7 (25–72)	27 (100%)	7 days	13/14 (93%) 12/13(92%)
[12]	Chang Gung Memorial Hospital, Taiwan	13 (31%)	58.6 (35–90)	13 (100%)	7 days	7/7(100%) 5/6(86%)
Lee et al., 2007	Chonnam National University, Toronto	16(44%)	47.1 (24–69)	9 (56%)	7 days	8/8 (100%) 10/10 (100%)
[13]	Madurai Medical College, India	4(25%)	50 (40–68)	4 (100%)	7 days (for EEA approach) and followed by craniotomy 2 weeks later	1/1(100)% 3/3(100%)
[14]	Nishi-Kobe Medical Center, Japan	12 (67%)	41(19–73)	9 (75%)	7 days	3/3 (100%) 6/6 (100%)
[15]	Kyungpook National University, Korea	12 (50%)	49 (16–74)	12 (100%)	7 days	8/8 (100%) 3/4 (75%)
[16]	Hanyang University Guri Hospital, Korea	29 (27.5%)	42.4 (25–68)	24 (100%)	48 h	16/19(84.2%) 5/8 (62.5%)
[17]	Salford Royal NHS England, UK	20(38.7%)	55 (n/a)	28 (91%)	48 h	11/11(100%) 9/9(100%)
[18]	University of California, San Francisco, USA	32(34%)	49 (10–79)	31(97%)	72 h	12/13(92%) 19/19 (100%)
[19]	Korea University Guro Hospital, Korea	41(29%)	52 (26–77)	35(85%)	7 days	24/24 (100%) 10/11(93%)
[9]	Vanderbilt University Medical Center, USA	14(44%)	58 (42–63)	26(81%)	48 h	7/11 (63.6%) 18/23 (78%)
[20]	Kocaeli University, Turkey	9(36%)	43 (18–72)	24 (86%)	7 days	14/14 (100%) 13/14 (92%)

**Table 2:** Demographic Characteristics of Included Studies, Surgical versus Conservative Intervention

Study	Institution, Country	Sample Size (%, female)	Mean Age of Cohort
[21]	Southern General Hospital, Glasgow, Scotland	Cons:6 Sx:9	Cons:52 Sx:52
[4]	Escola Paulista de Medicina, Sao Paolo, Brazil	Cons:12(41%) Sx:5(20%)	Cons:46 Sx:34
[22]	Hospital Universitario Puerta del Mar, Spain	Cons:4 Sx:5	52.4
[5]	Queen’s Medical Center, UK	Cons:20(20%) Sx:10(30%)	Cons:54 Sx:47
[23]	Queen Elizabeth Hospital, UK	Cons: 18(39.4%) Sx:15	Cons:54 Sx:51
[11]	Newcastle University Teaching Hospitals, UK	Cons:18(50%) Sx:27(30%)	Cons:46 Sx:51
[24]	Timone Hospital, Marseille, France	Cons:25(40%) Sx:19(37%)	Cons:55 Sx:50
[25]	South Australian Institute of Opthalmology, Adelaide, Australia	Cons:4(25%) Sx:18(28%)	Cons:47 Sx:54
[19]	Yeungnam University Hospital, Korea	Cons:32(38%) Sx:11(27.3%)	Cons:60 Sx:68
[8]	Mayo Clinic, USA	Cons:18(34.5) Sx:69	50.9
Culpin et al., 2016	Sheffield Children’s Hospital, UK	Cons: 4 (50%) Sx: 5 (40%)	Cons:15 Sx:15
[26]	Reference Center for Rare Pituitary Diseases, France	12 (50%)	49 (16–74)
[17]	Salford Royal NHS England, UK	Cons:11(37%) Sx:(40%)	Cons:53 Sx:56
[27]	National Hospital for Neurology and Neurosurgery, London, UK	55(34%)	53
[28]	University Hospital Aintree, London, UK	Cons:22(36.4) Sx:33	58

### Study selection process

Search results were screened against title and abstract by two reviewers. Points of disagreement were resolved by consultation with a third author serving as arbitrator until consensus was reached. Full texts were then screened to determine suitability for inclusion in the final review. The references of all included studies were examined to identify additional studies that may have been missed during initial screening for inclusion.

### Data extraction

Using standardized pro-forma, the following variables were extracted from each study: author and year of publication, study sample size, mean age, gender, timing of intervention relative to the onset of PA, visual acuity and/or field deficits, and ophthalmoplegia. The primary outcome of interest was visual recovery as measured by subjective postoperative patient symptomatology (improvement versus lack of improvement) and/or quantifiable measure of visual acuity via improved versus non-improved Snellen chart examination. Secondarily, we surveyed the literature in order to gather information regarding our second outcome of interest: visual recovery following conservative versus surgical management of PA.

### Data extraction and statistical analysis

Descriptive statistics were performed using Microsoft Excel 2019 (Redmond, WA, USA). Baseline characteristics along with outcomes of interest are presented as mean ± standard deviation for continuous outcomes, and counts and proportions for dichotomous, ordinal, and categorical variables. Quantitative meta-analysis was performed according to the Mantel–Haenszel method using Review Manager v5.4 (Nordic Cochrane Centre, Cochrane Collaboration, Copenhagen, Denmark). Comparisons made included pre- to post-operative changes in vision outcomes as stratified by timing (i.e., post-operative day (POD) 7 versus < POD 7). Additionally, surgical outcomes were compared to those obtained when conservative management was pursued. Odds ratios (ORs) and pooled 95% confidence intervals (CIs) were calculated to assess for the effect size of timing of surgical interventions and intervention modalities on primary outcomes. Results were presented as forest plots, representing ORs, relative weights, and 95% CIs. Heterogeneity across studies was evaluated using the Chi-square, I^2^ and τ^2^ tests. When I^2^ ≥ 50%, indicating substantial heterogeneity, a random-effects model was used. Alternatively, when I^2^ < 50%, indicating relatively less heterogeneity across studies, a fixed-effects model was used. Throughout all analyses, *p* < 0.05 was defined as the threshold for statistical significance. Review Manager provided funnel plots specific to each outcome as a representation of the risk of bias and the relationship between cohort size and effect size.

## Results

### Study selection and characteristics of included studies

**Fig. 1:**
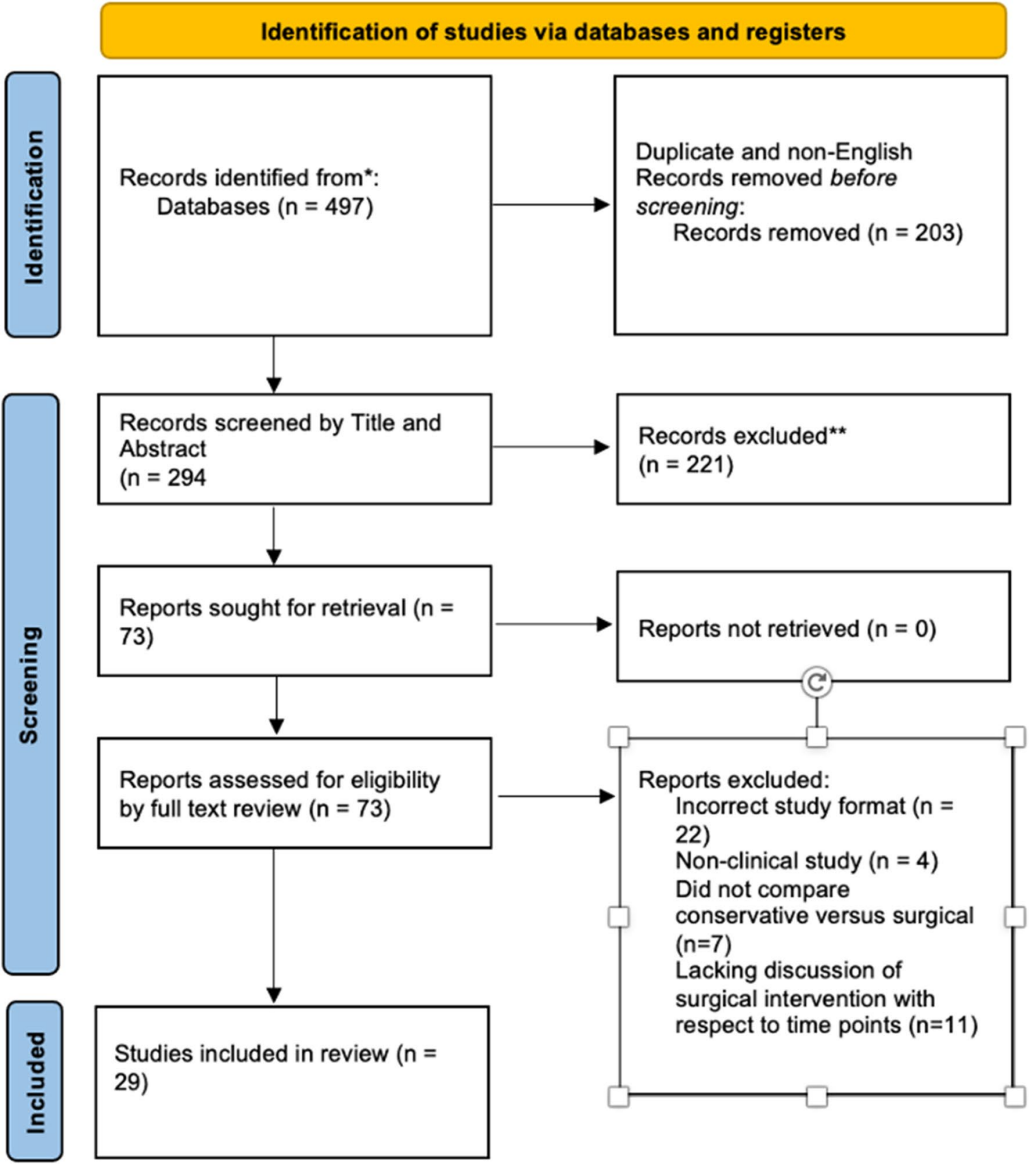
PRISMA flow diagram detailing study selection process

Of 294 unique search results, 44 studies focused on the timing of intervention for PA and met criteria for inclusion in full-text review (Fig. 1) [29–37]. After strict application of pre-defined inclusion and exclusion criteria, 29 studies remained eligible for review of vision outcomes follow pituitary apoplexy. Sixteen studies reported results with a focus on timing of surgical intervention, while 15 studies compared conservative versus surgical management of PA (two of the 29 studies were included in both sub-analyses). The most common reasons for exclusion were failure to report an overall rate for improvement in visual function [in other words, a total for all types of visual deficits combined] (*n* = 7), unclear delineation between intervention timepoints for each patient (*n* = 6), and failure to report the age and sex composition of the cohort (*n* = 4). The most common nations represented among the authors of these 29 studies included the UK (*n* = 8), USA (*n* = 4), and Korea (*n* = 4). The overall quality and risk of bias of included studies, according to the Newcastle–Ottawa Scale, was moderate.

### Patient demographics and interventions assessed: timing of surgical intervention

There was a total of 322 patients across the 16 [9–12, 14–18, 20, 38–42] studies included in the analysis of outcomes by timing of surgical intervention. With respect to sex, 83.6% (*n* = 269) of patients identified as male. The average ages of the patients included in the overall cohort ranged from 41 – 58.6 years (range: 10 – 90). Across all studies, most patients (range – min, max: 56%—100%) presented with a visual deficit. All patients in the cohort underwent surgical intervention for pituitary apoplexy, most commonly via the endoscopic endonasal approach (EEA), and those who underwent conservative management were excluded from this analysis. Surgical intervention was compared before versus after the 7-day timepoint in 12 studies, while other intervention timepoints of interest included 48 h and 72 h, in three studies and one study, respectively.

### Comparative assessment of interventions by timing

Across all studies, a majority (*n* = 274, 91.9%) of patients for whom preoperative visual status was reported presented with preoperative visual field deficits. There was no significant difference in baseline visual function deficits between patients who underwent surgery prior to versus after 7 days. The cohort comprising the comparison for the 7-day timepoint was the largest of the three comparison cohorts (220 patients) included in the present study. Furthermore, 28 patients underwent surgical decompression of the sella prior to 48 h and an additional 28 patients underwent decompression post-48 h (across the three studies focused on the 48-h timepoint). Finally, Rutkowski’s 2017 study was the only to assess outcomes prior to versus after intervention at the 72 h timepoint. Almost all patients in this study exhibited improved vision post-decompression, including 19/19 (100%) in the post-72-h cohort. Only one patient experienced worsened vision function 12/13 (92%), and this patient underwent surgery prior to 72 h.

**Fig. 2:**
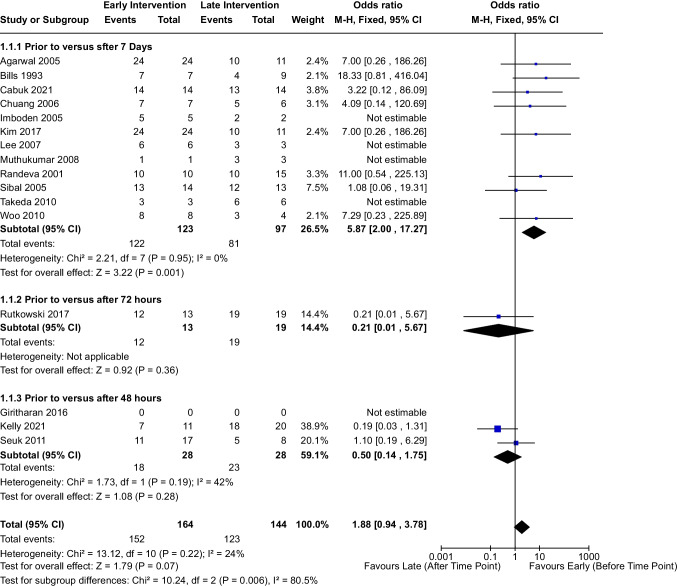
Forest plot demonstrating rates of improvement versus stabilization/decline in visual function following PA prior to versus after each designated time point. Values are represented as OR and 95% confidence intervals. The overall I.^2^ = 31% across all studies indicates homogeneity and minimal heterogeneity. The overall effect of timing of intervention appears to be insignificant, as noted by *p* > 0.05

On meta-analysis using the Mantel–Haenszel method, there was a significant difference (Fig. 2) in “absence of visual decline”, meaning stabilization or improvement of visual function pre-intervention, between patients who underwent surgical decompression before 7 days as compared to after seven days (OR 5.87, 95% CI [2.00, 17.27], I^2^ = 0%, *p* = 0.001). However, this trend was not noted for the studies investigating the 48-h (*p* = 0.28) or 72-h (*p* = 0.36) intervention timepoints.

### Patient demographics among studies comparing conservative and surgical interventions

There was a total of 288 patients across the 15 [4, 5, 8, 11, 17, 19, 21–28, 43] studies comparing surgical versus conservative management of pituitary apoplexy. The average ages of the patients included in studies featured in this sub-analysis ranged from 34 – 68 years.

### Comparative assessment of interventions by type (conservative versus surgical)

**Fig. 3:**
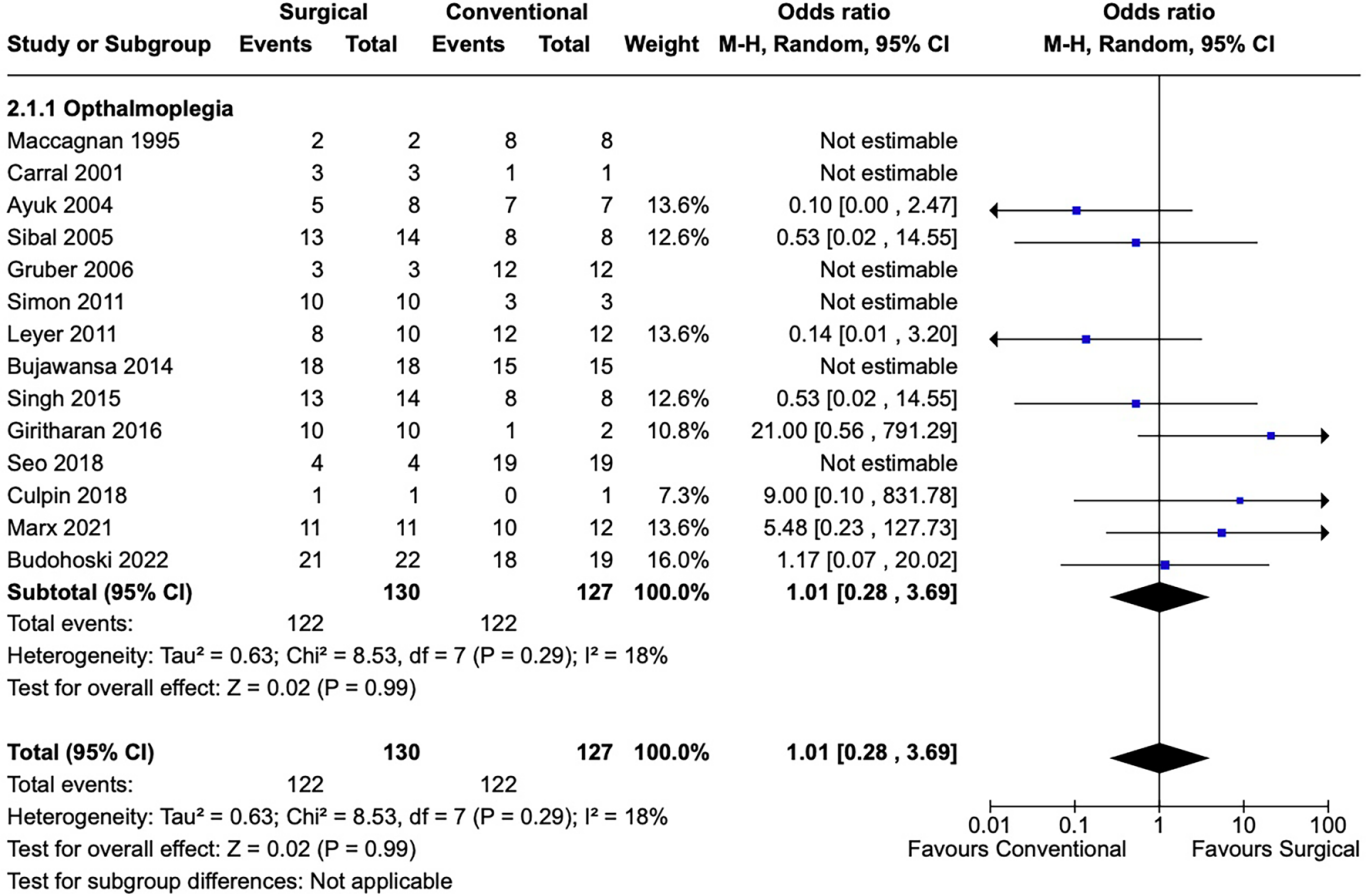
Forest plot demonstrates rates of ophthalmoplegia observed in conservative versus surgical management of pituitary apoplexy

**Fig. 4:**
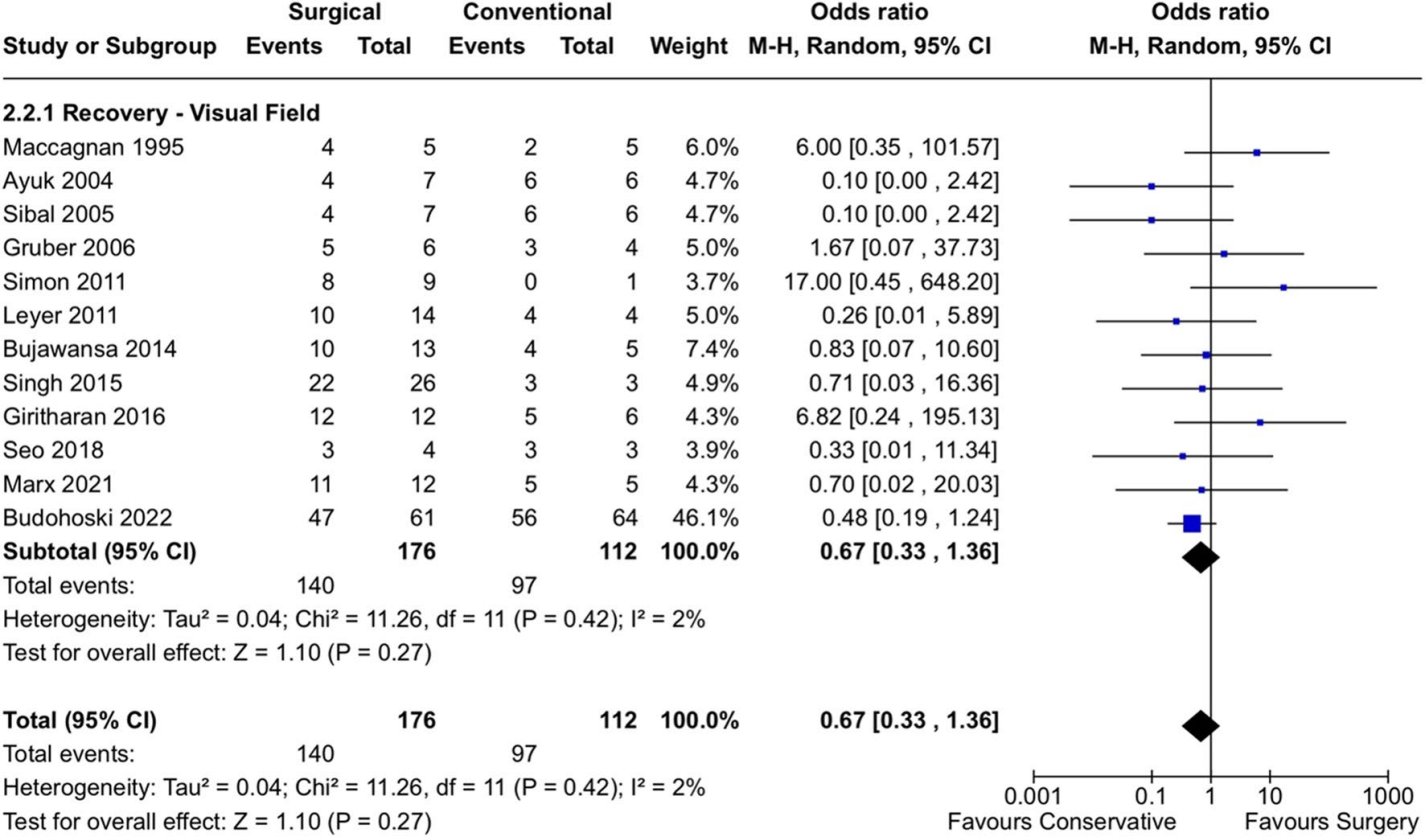
Forest plot demonstrating rates of visual field deficits observed in conservative versus surgical management of pituitary apoplexy

**Fig. 5:**
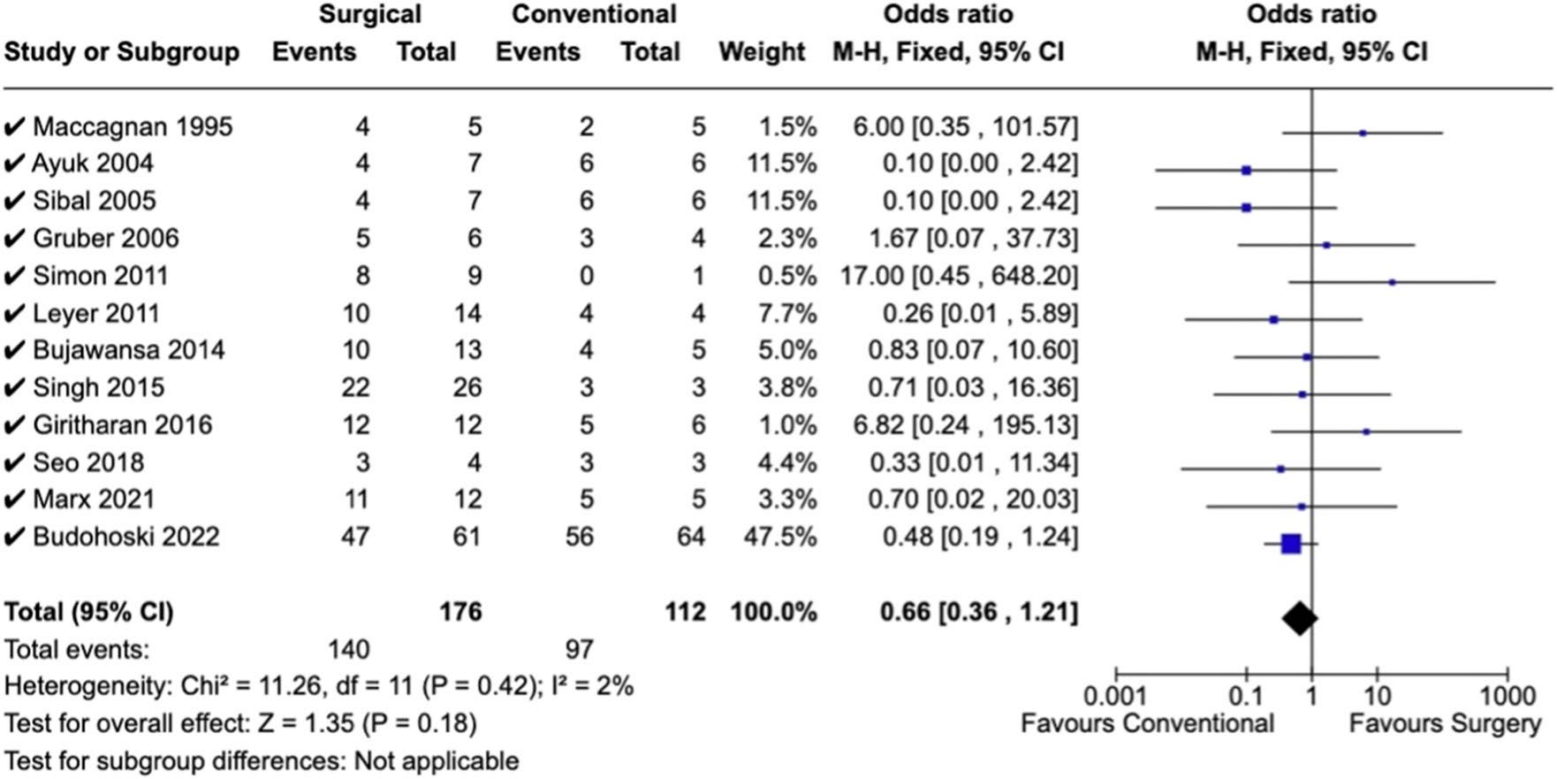
Forest plot demonstrating rates of “good” visual acuity obtained following conservative versus surgical management of pituitary apoplexy

Overall odds for recovery of visual deficits were comparable between conservative and surgical treatment cohorts across all studies and deficit categories reported. For example, although the OR for recovery from ophthalmoplegia and/or cranial nerve palsy was 0.91 [0.36, 2.31] for the surgical relative to conservative group (Fig. 3), this result was not statistically significant (*p* = 0.84). As I^2^ = 18%, heterogeneity across studies appears to have been low and is less likely to have influenced results. Similarly, results were equivocal with respect to recovery of visual field (OR 0.66 [0.36, 1.21], I^2^ = 2%, *p* = 0.18) and visual acuity (OR 0.63 [0.26,1.51], I^2^ = 0%, *p* = 0.30) for patients who underwent surgical intervention versus conservative treatment (Figs. 4 and 5).

## Discussion

In the present study, timing of intervention for acute, severe, symptomatic pituitary apoplexy was found to be predictive of visual function recovery at only the 7-day timepoint, as has been reported by previous studies [18, 44, 45]. However, no significant difference in post-operative visual outcomes was found when comparing interventions performed prior to versus after the 48 h or 72 h timepoints. Ultimately, this suggests that no further optimization of outcomes is obtained by taking PA patients to surgery “as soon as possible”. Nonetheless, drawing upon multiple studies that have been published over recent decades, operating within the first week does appear to afford superior vision outcomes when compared to “late” interventions (operating at > 7 days beyond the onset of apoplexy).

Furthermore, surgical decompression and conservative management appear to be effective treatments for pituitary apoplexy. Neither demonstrates superiority when assessing for ophthalmoplegia or cranial nerve deficits, visual field testing, or visual acuity [45]. Previously, reports have suggested that initiating conservative management is indicated when symptoms are mild at onset, but that severe visual deficits warrant emergent surgical decompression of the pituitary gland [11, 28]. Thus, both interventions can be effective when selected on a patient-to-patient basis. Ultimately, this is consistent with the results of the present meta-analysis. Nonetheless, since the 1970s, reports have described spontaneous recovery and remission of hyperpituitarism in PA, citing conservative management as instrumental to the successful outcomes obtained [21, 46, 47]. Eventually, this practice ascended to become the first-line treatment for severe PA at multiple pituitary centers worldwide [45]. Simultaneously, others have argued that surgical intervention should be automatically performed for patients demonstrating any acute onset neurological deficits [45].

Ultimately, the present analysis confirms that the standard-of-care management of PA has shifted over the decades from “surgical emergency” (take to OR immediately) to surgically urgent (< 48 h) to the present trend in care: surgery within 7 days or treatment with conservative management in patients with mild deficits. At present, the precise timing of surgery appears to be surgeon specific and training-dependent. In other words, preferences may be experience-dependent as well as based on the acuity and severity of symptoms, tumor size, histopathology, and secretory function of the tumor. All things considered, the present manuscript is intended to provide guidance for the subacute management of patients who develop this condition, as most cases will likely lie somewhere between clearly emergent (meriting immediate surgery) and non-emergent apoplexy.

### Perspectives on management of pituitary apoplexy over the decades

Clearly, management continues to represent an ongoing source of debate, and thus some skull base surgeons have taken a midline stance, choosing to tailor their approach to the specific patient. For example, some reserve surgery for patients with acute vision loss or other neurological deficits and manage patients with mild symptoms conservatively [45]. Even when these patients are treated conservatively, elective pituitary surgery remains an option for them down the line. Though simple in concept, implementing this treatment paradigm in reality is nuanced and not always straightforward. Most PA patients typically present somewhere along a spectrum ranging between the two categories: 1) mild, benign and 2) acute, emergent. As a result, many patients experiencing apoplexy contain hemorrhagic pituitary lesions that can be managed conservatively. However, some might argue that these patients should be taken to surgery [45].

To facilitate clinical decision-making, it can be helpful to reference a pituitary apoplexy grading scale. One such example is the Pituitary Apoplexy Score (PAS), which has previously been validated by Bujawansa and colleagues [28]. The PAS assigns points for diminished level of consciousness (GCS 15 = 0, GCS 2–7 = 4), visual acuity (normal = 0, reduced-unilateral = 1, reduced-bilateral = 2), visual field deficits (similar to visual acuity scoring), and ocular paresis (scored from 0–2 in a manner similar to visual acuity and visual field) [45]. One issue with this scale is that it does not necessarily provide precise quantification of deficits in mild forms of PA; as such, Jho and colleagues designed a modified Pituitary Apoplexy Grading System that simplifies grading into a scale ranging from 1–5. A score of “1” corresponds to absence of symptoms, while a score of “2” designates endocrinopathy only. Any visual acuity loss or visual field deficit is automatically assigned a score of 5, as is any patient presenting with a GCS too low for testing. The benefit of the Pituitary Apoplexy Grading System is that it is more sensitive for detection of mild symptomatology and enables a more straightforward indication-based assessment of visual function and overall neurologic status.

### United Kingdom guidelines for the management of pituitary apoplexy [48]

Currently, one of the most referenced guidelines for the treatment of PA are the U.K. guidelines developed by Wass and colleagues of the University of Oxford’s Churchill Hospital [48]. As stated within the guidelines, PA is a rare emergency most often associated with clinically nonfunctioning macroadenoma [11, 49]. Greater than three-quarters of the time, it is the first indication that a patient has an underlying, nonfunctioning pituitary macroadenoma. The condition is associated with a slight male predominance of approximately 1.6:1, and indeed the majority of patients in the current study were male [50, 51].

Because PA is often acute in onset and the first sign of an underlying pituitary lesion, diagnosis and intervention must proceed in a timely manner, but the reality is that the clinical picture can be conflated by uncertainty. For example, initial management is often dependent on the health care setting patients initially present to. In various settings, access to subspecialty opinions from endocrine, neurosurgical, and ophthalmologic providers may not be readily available [48]. When taken together with the fact that early versus late surgical decompression for PA is a topic of great debate, the waters surrounding optimal management of PA remain murky. It is thus not entirely clear which approach is the best for minimizing morbidity and mortality.

The underlying etiologies of the visual deficits associated with pituitary apoplexy – aside from optic nerve compression – are similar to deficits observed in cavernous sinus syndrome. Deficits are the result of sudden hemorrhage that results in rapid accumulation of blood in the confined space of the sella turcica and cavernous sinus, explaining why emergent alleviation of rapid bleeds is referred to as surgical decompression of the pituitary and its surrounding structures.

### Type of surgical intervention

Compression of cranial nerves III and VI can result in ophthalmoplegia, an extraocular muscle palsy that has been reported in 70% of cases of pituitary apoplexy [52, 53]. Of the cranial nerve palsies observed in apoplexy, CN III palsy is in fact the most common and represents approximately half [49]. When mass effect is exerted upon the optic chiasm, bitemporal hemianopsia will be a presenting sign/symptom – reported in approximately 75% of pituitary apoplexy patients with visual dysfunction [41]. Prior literature suggests that patients with mild visual deficits are candidates for conservative treatment and that ophthalmoplegia will resolve without surgical intervention in these cases [54]. Nonetheless, exceptions to this rule can arise and this is only reasonable management for patients with an overall clinical presentation that can be considered mild [24].

When permissible, conservative treatment is preferred because surgical decompression is associated with risks of any surgical procedure involving the pituitary gland: endocrinopathy, hemorrhage, CSF leak, and death [11]. Performing surgery for pituitary apoplexy is indicated when significant cranial nerve and/or ophthalmologic defects are detected on exam or the patient presents in or deteriorates into a low state of consciousness/mental status (indicating significant mass effect or elevated ICP). Altogether, our analysis suggests that, among studies claiming to adhere to treatment protocols along these lines, outcomes are roughly equivocal following conservative management and surgery. Both can be effective when patient selection is performed carefully and dictated by the results of a thorough, meticulous neurological examination and subsequent documentation of any deviations from the baseline exam [38].

### Timing of surgical intervention

Of course, management of pituitary apoplexy is not quite this simple: seeing this through is easier said than done, no single patient is alike, and like most pathologies of the CNS, management can be nuanced, and gray areas do exist. As previously mentioned, the seven-day window is generally considered the ideal timeframe within which to operate, and this recommendation is supported by the U.K. guidelines [48]. Despite this being a commonly respected recommendation, previous studies have found that visual field and acuity outcomes before and after the seven-day timepoint are comparable; however, this does not appear to be the case for patients with preoperative ophthalmoplegia. In fact, these patients experience ocular palsy recovery rates of roughly 30%-60%, far lower than ocular functional recovery rates observed when ophthalmoplegia is absent (70%-90%) [13, 24, 28]. This suggests that ophthalmoplegia is a sign indicating that more urgent surgical intervention is warranted.

Regardless of timing, it is essential that fluid resuscitation and possibly corticosteroids be administered in any severe presentation that is slated to undergo surgery [53]. PA patients should be stabilized through monitoring of electrolyte levels and managing pituitary hormone imbalances (such as administering hydrocortisone to prevent circulatory collapse) [8]. Furthermore, the PAS grading system has emerged as a useful tool that can help neurosurgeons decide whether to intervene operatively (after the patient is stabilized). The PAS, introduced by Reddy and colleagues, offers a method for deriving a calculated measure of the severity of a given PA presentation, and several retrospective studies have suggested that it is useful in predicting whether conservative versus surgical management should be pursued [28, 55, 56]. Further investigations are merited so that the validity of this tool can be verified for clinical application.

### Recent updates regarding treatment of pituitary apoplexy

In 2021, Shepard and colleagues received attention after publishing their single-center retrospective study on clinical and radiologic outcomes of pituitary apoplexy by management strategy (conservative versus early surgical intervention) [7]. The authors reported that most PA cases can be successfully managed with conservative treatment, particularly when patients present with minimal visual defects such as incomplete bilateral temporal hemianopia or partial cranial neuropathy). However, as pointed out by Wang and colleagues, early surgery was not categorized on the basis of what would typically be defined as “early” (< 48 h) [57]. Instead, the authors defined early surgery as surgery within 1 week of PA diagnosis. The study’s findings, that surgery should be performed within 1 week for patients with severe visual field deficits and ocular palsies, are therefore consistent with recommendations offered in the literature. With respect to the surgery versus conservative treatment debate, patients who underwent surgery in Shepard’s study had larger PA volumes and tumor diameters, on average, than patients in the conservative group. Coupled with the finding that patients in the early surgical group had larger tumors on average, Shepard reported that patients with deteriorating vision and/or severe visual impairment were more likely to undergo early surgery [7]. The difficulty in interpreting these results is that it is difficult to capture what role initial presentation had in ultimate prognosis. In other words, were ultimate outcomes influenced more by severity of presentation or the course of treatment pursued (surgical versus conservative)? In response to this question, Shepard and Jane Jr. acknowledged that management of PA remains a highly controversial topic. Furthermore, they reiterated that their series was meant to illustrate their institutional practice for management of patients with PA: conservative treatment with high-dose steroids and close neurologic/ophthalmologic monitoring, and that their goal was to supply evidence indicating that successful outcomes can be obtained through conservative management of PA, indicating that it is not universally a condition that merits emergency surgery [7, 58]. Furthermore, Shepard and Jane Jr. emphasized that all patients with normal visual function were managed conservatively, and that there were no cases of visual function decline in these patients. Of patients in whom conservative management failed, only three had transient decline in visual function that improved following surgery. By contrast, all patients who underwent early surgery for visual acuity or field deficits (or ophthalmoplegia) in the setting of PA experienced subsequent visual decline. Though their outcomes were worse, Shepard and Jane Jr. argue that this is because early surgery should be reserved for the most severe cases of PA; the cases that by default will exhibit more advanced deficits post-intervention (as this is further proof of the urgent and/or emergent nature of their presentation). Otherwise, Shepard and Jane Jr. suggest that conservative management is effective for non-severe cases of apoplexy and that the challenge that has hindered the development of one standard guideline for treatment of PA is that apoplexy is a spectrum of diseases as opposed to a single clinical entity. Finally, they acknowledge that early surgery may be reserved for select cases in which patients present with severe visual acuity loss or visual field defects, but that the majority of cases can be managed conservatively. When surgical intervention is indicated, there is no evidence that performing surgery < 48 h following apoplexy provides any advantage over intervening < 1 week after onset. Accordingly, multiple reports within the current literature consider any surgery performed within 1 week as “early” surgical intervention [7].

Finally, another relevant question that has emerged is whether or not outcomes differ depending on whether or not the pituitary tumor is a functioning or non-functioning adenoma. At present, the literature indicates that there is little to no difference in outcomes between patients experiencing apoplexy who harbor functioning adenomas as compared to those who have underlying non-functioning adenomas [59]. Of course, the vast majority of patients with apoplexy secondary to pituitary macroadenoma experience apoplexy as the first sign of their underlying pituitary mass. If not for the apoplexy, the pituitary lesion would continue growing in an asymptomatic manner until becoming symptomatic due to mass effect. Because non-functioning pituitary adenomas are non-secretory and therefore do not directly cause hormonal imbalances or systemic side effects (hyperprolactinemia, acromegaly, Cushing’s disease, etc.), they can grow insidiously until they become quite large. Oftentimes, their clinically silent nature means that they will grow until apoplexy occurs as the first sign of their presence. By this time, they are often > 10 mm in diameter, and this explains why patients experiencing pituitary apoplexy most commonly harbor non-functioning pituitary macroadenomas. Nonetheless, although far less common, it is possible for apoplexy to occur in the setting of functioning pituitary macro- and microadenomas. Although these lesions technically possess active secretory endocrine activity, they usually do not manifest with noticeable symptoms or hormonal side effects prior to apoplexy; when apoplexy occurs in the setting of a functioning pituitary adenoma it is often the first presenting sign, just as is the case for nonfunctioning adenomas. According to Nakhleh and colleagues, no differences in endocrine or neuro-opthalmic outcomes were observed between patients with functioning and nonfunctioning adenomas by final follow-up in their retrospective study spanning over two decades [59]. Just as clinical course did not vary by adenoma subtype, consistent results were obtained for patients who underwent conservative and surgical management [59].

### Limitations

We acknowledge several limitations to the present study. First and foremost, its retrospective nature as a study featuring heterogeneous data makes it difficult to rule out potential sources of bias. Therefore, the results would benefit from validation through large, multi-institutional randomized controlled trials or registry database studies to investigate the influence of intervention timing and modality on both visual and non-visual clinical outcomes.

Additionally, the analysis was unable to determine specific recovery rates based on the type of visual deficit due to the heterogeneity in the manner by which visual deficits were reported. Unfortunately, subgroup analyses of visual recovery based on the type of presenting visual deficit could not be conducted. Visual recovery was mainly assessed qualitatively, relying on subjective patient-reported improvement in vision as the primary outcome measure. Future investigations should include objective ophthalmologic testing methods such as optical coherence tomography (OCT) and perimetry to quantify improvements in visual deficits before and after surgical intervention. Positive-publication bias is another potential concern, as published data may be more likely to report positive outcomes rather than negative ones in relation to postoperative vision changes. This bias could artificially inflate the reported visual recovery rates. Future prospective or registry studies should aim to evaluate the effects of timing of surgical decompression on clinical outcomes in patients with pituitary apoplexy. Finally, it is unclear whether the population of patients included in this study are representative of the general population of apoplexy patients. The main factor suggesting this is that males comprised approximately 84% of the study population, even though previous studies have consistently reported that apoplexy demonstrates a male predominance ranging from 1.1 to 2.3/1 [60–62]. The patients included in the present study exhibit a fairly extreme male predominance, suggesting that the generalizability of the study population is a potential limitation of our meta-analysis.

In summation, while this meta-analysis provides a foundation for future prospective or registry studies, it is important to acknowledge the limitations of the available literature regarding timing of intervention, intervention modality, and functional recovery in patients with pituitary apoplexy. The analysis supports the benefits of surgical intervention, but further investigation is needed to determine whether there is an optimal timing cutoff that must be adhered to when managing pituitary apoplexy that would prevent potentially devastating ocular consequences.

## Conclusion

In the present study, timing of surgical intervention for pituitary apoplexy was only found to be predictive of visual function recovery when surgery commenced prior to the 7-day timepoint, as has been reported by previous studies. Ultimately, this suggests that timing of intervention may have a significant impact on one of the most serious consequences of PA: visual field loss and/or ocular dysfunction. However, it does not appear that the specific time the patient is taken to surgery (within the 7-day time frame) influences outcomes. Furthermore, with respect to intervention modality, it appears that both surgical and conservative management can prove effective in carefully selected patients. Overall, the specific treatment modality selected and the timing of treatment appear to be less important than the immediate administration of IV corticosteroids once the diagnosis of apoplexy is confirmed. Thus, urgent medical resuscitation and/or management is paramount, but the specifics of subsequent treatment may not be as pivotal as they were long thought to be. In other words, any cases of circulatory shock should be addressed from a medical standpoint, as should corticotropic deficiency because secondary adrenal insufficiency can prove lethal if not promptly addressed. Otherwise, the literature is lacking official guidelines to assist patient selection for surgical intervention and thus future prospective and multicenter studies are warranted.

## Abbreviations

CN: Cranial nerve
CSF: Cerebrospinal fluid
EEA: Endoscopic endonasal
PA: Pituitary apoplexy
